# Trends of low physical activity among Iranian adolescents across urban and rural areas during 2006–2011

**DOI:** 10.1038/s41598-020-78048-0

**Published:** 2020-12-07

**Authors:** Parisa Amiri, Parisa Naseri, Golnaz Vahedi-Notash, Sara Jalali-Farahani, Yadollah Mehrabi, Najmeh Hamzavi-Zarghani, Fereidoun Azizi, Farzad Hadaegh, Davood Khalili

**Affiliations:** 1 Research Center for Social Determinants of Health, Research Institute for Endocrine Sciences, Shahid Beheshti University of Medical Sciences, Tehran, Islamic Republic of Iran; 2 Department of Biostatistics, Faculty of Paramedical Sciences, Shahid Beheshti University of Medical Sciences, Tehran, Islamic Republic of Iran; 3 Department of Epidemiology, School of Public Health and Safety, Shahid Beheshti University of Medical Sciences, Tehran, Islamic Republic of Iran; 4 Endocrine Research Center, Research Institute for Endocrine Sciences, Shahid Beheshti University of Medical Sciences, Tehran, Islamic Republic of Iran; 5 Prevention of Metabolic Disorders Research Center, Research Institute for Endocrine Sciences, Shahid Beheshti University of Medical Sciences, P.O. Box: 19395-4763, Tehran, Islamic Republic of Iran

**Keywords:** Disease prevention, Health policy, Public health, Quality of life, Weight management

## Abstract

It is well documented that physical inactivity is related to weight gain and a whole host of chronic diseases. This study investigated trends of low physical activity among Iranian adolescents in urban and rural areas between 2006–2011. A total of 12,178 adolescents, aged between 15 and 19 years, participated in National Surveys of Risk Factors for Non-Communicable Diseases. Data on physical activity was obtained using the global physical activity questionnaire. A complex sample survey and multinomial logistic regression were used to model physical activity levels. The percentage of adolescents who had low levels of physical activity increased from 2006 to 2011 in both urban and rural areas. Low and moderate levels of physical activity were lower in rural girls as compared with urban girls, with a prevalence ratio of 0.59 (95% CI 0.47–0.74) and 0.59 (95% CI 0.47–0.74), respectively. The corresponding values for boys residing in rural areas compared with boys in urban areas were 0.56 (95% CI 0.43–0.75) and 0.60 (95% CI 0.48–0.74), respectively. The adolescents' lifestyles showed an increasing trend for physical inactivity in both genders; however, in rural areas, only girls had a rising affinity for a sedentary lifestyle throughout the 2006–2011 years.

## Introduction

Based on the global statistics, physical inactivity has increased dramatically in adolescents aged 13–15 years in more than one hundred countries over the past decades^1^. Nowadays, the fast-growing attraction for children and adolescents to view TV/film screens and play video games has become prevalent in both developed and developing nations^1–3^. According to the World Health Organization (WHO), recommended daily physical activity for children and adolescents aged 5–17 years is at least 60 min of moderate- to vigorous-intensity physical activity per day^4^. In addition, "low physical activity" in young childhood is defined as not achieving a minimum of at least 600 MET-minutes per week. A recent WHO report indicated that over 80% of adolescents aged 11–17 years had low physical activity^5^.

Furthermore, in a study conducted by Subhi, only less than one-fifth of children in early adolescence met the adequate daily physical activity requirements in ten Eastern Mediterranean region countries^6^. The results of a systematic review in Iran revealed that the prevalence of physical inactivity and sedentary lifestyle among adolescence is high^7^, and over 45% of Iranian school children do not have adequate levels of physical activity^8^. There is a whole host of evidence to demonstrate that insufficient physical exercise in youth can be a crucial indicator of an unhealthy lifestyle, as well as weight disorders, which can lead to major chronic diseases such as diabetes type 2, cardiovascular diseases and cancers^9–12^.

It is well known that adolescence is a critical period of life for shaping healthy behaviors that affect an individual's future habits and wellbeing. Adolescents experience essential physical and psychological changes that place them at an increased risk of becoming overweight. Evidence shows that as children near teens, they decrease physical activity and engage in more sedentary activities in recent years^13^. Although the association between low physical activity and excessive weight gain in the early years of life has been well documented^14,15^, further solid evidence has complicated matters by disclosing that even underweight children do not meet desirable levels of physical activity^16–18^. Findings from a study evaluating adolescents' physical activity in the United States also showed that both underweight and overweight teens had low levels of physical activity^16^.

Additionally, the results of a large cross-sectional study conducted on school-aged children in different Eastern Mediterranean countries revealed that the proportion of children and adolescents who were physically active was surprisingly highest in the overweight subjects-followed by the normal and underweight groups^6^. Intriguingly, a study conducted in Iran also showed no significant correlation between time spent on different physical activities and age-specific BMI in the studied population^19^. On the other hand, results of a national survey evaluating a representative sample of Iranian children and adolescents indicated that compared to normal weight children, both underweight and obese children were more likely to be inactive^20^; the prevalence of physical inactivity observed in children from all weight groups emphasizes the importance of physical activity monitoring in all Iranian children, not only obese ones. In addition to weight status, gender-specific patterns were observed in several previous studies^2,21–23^. Existing data on the global physical activity levels of adolescence reveals that boys are more active than girls^1^. Hence, gender group as well as weight status were considered to investigate trends of low physical activity in the current study.

A growing body of evidence has asserted that apart from personal factors mentioned, environmental, cultural, economic, and technological development, as well as transportation and urbanization, has notably affected the daily physical activity practices of individuals^12,24–26^. Urbanization involves the growth of cities and migration from rural areas; moreover, behavioral changes will also occur through the often accompanying industrialization and economic development^27^. One study reported a complex association between dietary transition, urbanization and increased public transport and decreased levels of physical activity- and therefore-a rising prevalence of obesity in developing countries^28^. Besides, the findings of a cohort study from Italy revealed linear associations between physical activity and obesity with increasing urbanization^24^, concurring with other studies that realized rural adolescents spend more time on outdoor activities than urban youths^24,29^. A recent national survey revealed that although physical activity levels are low among Iranian children and adolescents, students residing in rural areas spend more time being physically active^30^. We may conclude from this analysis that the type of environment can influence lifestyle patterns, including daily physical activity, especially during the early years of life.

Iran is a country with an approximately 75 million population (49% female), two-thirds of which reside in urban areas. According to the results of a national survey, Iranian children and adolescents living in rural and urban areas have different levels of physical activity^8^. However, related studies and national reports are cross-sectional and have focused mainly on physical activity and its determinants among Iranian adolescents^8,12^. The main objective of this study was to examine the trend of low physical activity in Iranian adolescents aged 15–19 years, residing across urban and rural areas using data from the national Surveillance of Risk Factors of Non-Communicable Diseases (SuRFNCD) from 2006 to 2011.

## Methods

### Study population

This study was conducted within the framework of the national Surveillance of Risk Factors of Non-Communicable Diseases (SuRFNCD) conducted through 2006–2011; this was based on the WHO STEP-wise approach to Surveillance (STEPS) for WHO member countries as a simple, standardized method for collecting, analyzing and disseminating data (available at https://www.who.int/ncds/surveillance/steps/en/). This approach encourages countries to collect small amounts of useful information in a regular and continuous manner. The STEPS instrument covers three different steps of risk factor assessment, including questionnaire, physical exam and biochemical measurements. In Iran, the first two steps were implemented each year, except in 2010, when the survey was not carried out due to administrative and financial constraints. A representative sample of urban and rural individuals was selected based on a multistage random cluster sampling method; more details of the SuRFNCD have been reported previously^31^. The surveys received ethics approval from the Center for Disease Control in Iran. All participants gave informed consent. Interviewers were trained on all details of the survey in a 1-day workshop in Tehran. The age range of the surveys is usually 15–65 years—however, for this current analysis—a total of 12,178 adolescents aged 15–19 years were considered, including 2006 (n = 2595), 2007 (n = 2897), 2008 (n = 2950), 2009 (n = 2827) and 2011 (n = 909). Data on variables including sex, age, residential area, physical activity, and general obesity were analyzed.

### Measurements

The global physical activity questionnaire (GPAQ) endorsed by WHO was used as a standard questionnaire for measuring physical activity. The translation of the second version of GPAQ has been used for the assessment of physical activity in SuRFNCD studies with acceptable reliability and validity^32^. In these surveys, demographic information such as sex, age, province and area were also documented.

The weight and height of participants were measured in light clothing without shoes, using a portable calibrated electronic weighing scale and a portable height scale, respectively^27^. Body mass index (BMI) was calculated based on the Quetelet formula (weight (kg)/height^2^ (m))^33^.

### Definition of terms

According to the GPAQ questionnaire, physical activity was categorized at three levels: (1) High: a vigorous-intensity activity that was performed at least 3 days a week, achieving a minimum of at least 1500 metabolic equivalent (MET) minutes per week, or 7 days of any combination of walking and moderate- or vigorous-intensity activities, achieving a minimum of at least 3000 MET-minute/week; (2) Moderate: ≥ 3 days of vigorous-intensity activity, at least 20 min/day, or ≥ 5 days of moderate-intensity activity (including walking) of at least 30 min per day, or five or more days of any combination of walking, moderate- or vigorous intensity activities, achieving a minimum of at least 600 MET-minutes per week and (3) Low: not meeting any of the above mentioned criteria^32^.

General obesity status was defined based on BMI z-score, which was determined using a child's age, sex, BMI (kg/m^2^), and an appropriate reference standard. For adolescents aged 15–18 years, weight status was defined as follows: − 2SD ≤ underweight < SD, 1SD ≤ normal < 2SD, 2SD ≤ overweight < 3SD and obesity ≥ 3SD. For individuals aged 19–20 years, BMI was categorized into four groups, including underweight (BMI < 18.5), normal weight (18.5 ≤ BMI < 25), overweight (25 ≤ BMI < 30) and obese (30 ≤ BMI). In this study, underweight and normal weight participants were considered as the non-obese group, while overweight and obese individuals were considered as the obese group.

### Statistical analysis

Complex sample survey analysis was used to provide representative estimates of the Iranian population in 2006, 2007, 2008, 2009, and 2011. According to the 2011 national census of Iran, sampling weights were generated based on sex and area (rural/urban) strata.

Data from 2006 to 2011 SuRFNCD were pooled for analysis. Continuous variables are presented as mean ± standard error and categorical variables are expressed as frequencies (%). Multinomial logistic regression was applied to model physical activity levels. Accordingly, physical activity levels were considered as the dependent variable and the years were included as a categorical variable. Corresponding values for each time point of the study were as follows: 0 for 2006, 1 for 2007, 2 for 2008, 3 for 2009 and 5 for 2011. Models 1^a^ and 1^b^ were adjusted for years. Model 1^a^ was stratified based on residential areas. In this model, sex and obesity status were considered as independent variables. Model 1^b^ was stratified based on sex categories and areas and obesity status were included as independent variables. Also, the interaction effects (sex × obesity status, area × obesity status) were examined in models 1^a^ and 1^b^, respectively. Prevalence ratios (PR) and 95% confidence intervals (CIs) were calculated for the models mentioned.

**Figure1:**
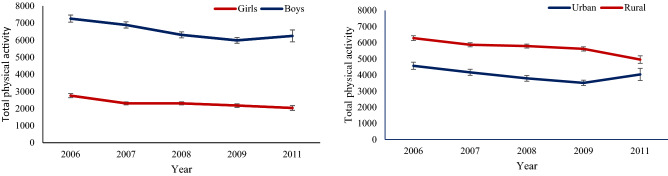
Trend of total physical activity (MET-minutes per week) of adolescents, aged 15–19 years: SuRFNCD 2006–2011. (**a**) Based on sex strata. *P* for trend in boys = 0.03; *P* for trend in girls = 0.02; *P*-interaction for sex/year = 0.3. (**b**) Based on residential areas. *P*-interaction for area/year = 0.25; *P* for trend in urban = 0.07; *P* for trend in rural = 0.04. Error bars shows standard error.

To assist the presentation and comprehension of the results, we illustrate the means of total physical activity throughout 2006–2011 surveys across sex strata and areas of residence in separate figures (Fig. 1a,b). For assessing the linear trend of total physical activity through the years, linear regression was fitted for sex and areas independently. In each model, the effect of year was reported as p for trend. Also, differences in means of total physical activity over the years across the sex strata and areas of residence were examined by modeling interaction terms (sex × year, area × year), respectively.

The unadjusted prevalence rates of low physical activity and their 95% CIs, based on sex/area categories over the years, were calculated. Temporal trends of low physical activity prevalence during 2006–2011 were assessed by linear regression as well. Analyses were conducted using STATA version 14^34^. *P-values* < 0.05 were considered statistically significant.

## Results

There were 12,178 participants, 5,588 girls (45.9%) and 6,590 boys (54.1%) for these repeated cross-sectional studies. Socio-demographic and anthropometric characteristics of the study population are presented in Table 1. The mean age of adolescents was approximately 17 years in each survey.

**Table 1:** Area-specific characteristics of adolescents aged 15–19: SuRFNCD2006–2011.

	Urban					Rural				
	2006 (n = 1580)	2007 (n = 1688)	2008 (n = 1797)	2009 (n = 1523)	2011 (n = 601)	2006 (n = 1015)	2007 (n = 1209)	2008 (n = 1153)	2009 (n = 1304)	**2011** **(n = 308)**
**Age** (years)	17.76 ± 0.02	17.60 ± 0.03	17.58 ± 0.03	17.58 ± 0.04	17.74 ± 0.05	17.83 ± 0.03	17.55 ± 0.03	17.55 ± 0.04	17.60 ± 0.03	17.61 ± 0.08
**Sex** n(%)										
Girls	693(43.9)	738(43.7)	800(44.5)	709(46.6)	327(54.4)	495(48.8)	547(45.2)	516(44.8)	600 (46)	163(52.9)
Boys	887(56.1)	950(56.3)	997(55.5)	814 (53.4)	274(45.6)	520(51.2)	662(54.8)	637(55.2)	704(54)	145(47.1)
**Physical activity** (% ± SE)										
High	47.30 ± 1.14	42.74 ± 1.11	40.25 ± 1.10	37.42 ± 1.19	41.41 ± 2.08	55.04 ± 1.38	53.75 ± 1.33	56.16 ± 1.36	54.73 ± 1.29	44.64 ± 2.85
Moderate	32.94 ± 1.19	32.68 ± 1.19	30.24 ± 1.13	32.81 ± 1.26	27.68 ± 1.97	25.95 ± 1.34	26.52 ± 1.30	22.52 ± 1.27	24.46 ± 1.24	26.89 ± 2.77
Low	19.74 ± 1.02	24.56 ± 1.06	29.49 ± 1.09	29.75 ± 1.19	30.90 ± 1.87	18.99 ± 1.18	19.72 ± 1.14	21.30 ± 1.20	20.79 ± 1.11	28.46 ± 2.63
**General obesity** (% ± SE)										
Obese	20.68 ± 1.01	21.50 ± 0.99	21.14 ± 0.97	22.80 ± 1.07	21.96 ± 1.68	15.46 ± 1.08	15.21 ± 1.02	15.50 ± 1.06	17.63 ± 1.05	17.05 ± 2.05
Non-obese	79.31 ± 1.01	78.49 ± 0.99	78.85 ± 0.97	77.19 ± 1.07	78.03 ± 1.68	84.53 ± 1.08	84.78 ± 1.02	84.49 ± 1.06	82.36 ± 1.05	82.94 ± 2.05

Age value is Mean ± SEM. Variables (except sex) are standardized according to sex.

Non-obese: underweight/normal.

Obese: overweight/obese.

Remarkably the percentage of adolescents with high physical activity decreased from 2006 to 2011 in both urban and rural areas, whereas the percentage of individuals with low physical activity rose. Moreover, based on obesity status from 2006 to 2011, the percentage of non-obese individuals decreased in urban and rural areas, whereas the percentage of obese individuals increased in both residential areas. Sex-specific characteristics of participants are shown in Table 1-Appendix. Results of multinomial logistic models are presented in Table 2.

**Table 2:** Prevalence ratio (PR) of low and moderate physical activity according to adolescents' (aged 15–19 years) residential area: SuRFNCD2006- 2011.

	Urban		Rural	
	PR for low(95%CI)	PR for moderate(95%CI)	PR for low(95%CI)	PR for moderate(95%CI)
**Model 1**^**a**^				
Girls	Reference	Reference	Reference	Reference
Boys	0.11*(0.09–0.14)	0.20* (0.17–0.25)	0.11*(0.10–0.14)	0.21*(0.18–0.24)
Non-obese	Reference	Reference	Reference	Reference
Obese	0.99(0.77–1.27)	1.01(0.80–1.27)	1.06(0.74–1.51)	0.97(0.68–1.36)

	Girls		Boys	
	PR for low(95%CI)	PR for moderate(95%CI)	PR for low(95%CI)	PR for moderate(95%CI)
**Model 1**^**b**^				
Urban	Reference	Reference	Reference	Reference
Rural	0.59*(0.47–0.74)	0.59*(0.47–0.74)	0.56*(0.43–0.75)	0.60*(0.48–0.74)
Non-obese	Reference	Reference	Reference	Reference
Obese	0.86(0.65–1.14)	0.83(0.62–1.09)	1.15(0.84–1.56)	1.16(0.89–1.51)

Low physical activity: lower than 600 MET-minutes per week, Moderate physical activity: at least 600 MET-minutes per week, High physical activity: at least 3,000 MET-minutes per week.

Models are adjusted for the year.

Reference: High physical activity in multinomial logistic.

*P* values < 0.001.

*P*-interaction for sex-obesity in low physical activity = 0.11; sex-obesity in moderate physical activity = 0.07; area-obesity in low physical activity = 0.5; area-obesity in moderate physical activity = 0.97.

*P-values < 0.05 were considered statistically significant.

After adjusting for years in model 1^a^, the prevalence of low and moderate physical activity for boys was lower than girls in both residential areas. In urban areas, the prevalence ratio of low and moderate physical activity for boys compared to girls was 0.11 (95% CI 0.09–0.14) and 0.20 (95% CI 0.17–0.25), respectively. The corresponding values in rural areas were 0.11 (95% CI 0.10–0.14) and 0.21(95% CI 0.18–0.24), respectively. However, obese adolescents did not show different patterns of physical activity, compared to their non-obese counterparts. In model 1^b^, the prevalence ratios of low and moderate physical activity for rural girls were 0.59 (95% CI 0.47–0.74) and 0.59 (95% CI 0.47–0.74) compared to urban girls. The corresponding values were 0.56 (95% CI 0.43–0.75) and 0.60(95% CI 0.48–0.74) in boys, respectively.

The mean of total physical activity in all the national surveys is shown for gender and residential area categories separately (Fig. 1a,b).

Accordingly to the survey, a significant decrease in total physical activity was observed among both genders and residential areas from 2006 to 2009 (*P* < 0.05); however, the mean total physical activity increased from 2009 to 2011 in boys from urban areas. In addition, there was no significant interaction between sex and years (*P* = 0.3) or between residential areas and years (*P* = 0.25).

Unadjusted prevalence of low physical activity based on sex and residential area from 2006 to 2011 is presented in Table 2—appendix.

In both genders and areas, the highest and lowest prevalence of low physical activity was observed in 2011 and 2006, respectively. The prevalence of low physical activity across sex and residential area categories showed that the trend of low physical activity from 2006 to 2011 increased significantly in girls in both areas and in boys residing in urban areas (*P* < 0.05).

## Discussion

This is the first national report that has analyzed and discussed physical activity trends in adolescents residing in urban and rural areas of an Eastern Mediterranean country. Our results indicate a rising trend of low physical activity in both genders living in urban areas and only in girls living in rural areas of Iran through the study period (Table 2—Appendix). Among different measurements, the lowest and highest prevalence of low physical activity were observed in 2006 and 2011, respectively (Table 2—Appendix); in other words, a positive trend towards a sedentary lifestyle seems evident in most adolescents. The low and moderate physical activity were less prevalent among rural, compared to urban adolescents and in boys, compared to girls in both residential areas. In both genders and residential areas, physical activity was independent of weight (Table 2).

The current rising trend of low physical activity among Iranian adolescents is in line with similar decreasing trends of physical activity among American boys and girls^20^. To the best of our knowledge, there is no other comparable longitudinal data regarding trends of physical activity in Iranian adolescents. However, there has been a high prevalence of physical inactivity documented in some cross-sectional studies that were conducted in different regions of Iran^12,35^. Based on a WHO report, the highest prevalence of physical inactivity was observed in the Eastern Mediterranean, the African and the Western Pacific regions (88%, 85%, and 85%, respectively)^1,36^. Moreover, a systematic review of studies conducted between 2006- 2012 in Arab countries showed an alarming prevalence of physical inactivity among children and adolescents, ranging from 65%- 91%^37^.

Based on the current results, we can conclude that an alarming positive trend of physical inactivity was observed in all geographical areas of Iran, which was more prevalent in urban—than in rural regions (Table 1). Existing data on the patterns of physical activity in rural and urban areas from different countries is limited and controversial. Despite similar geographical patterns of physical activity observed in the USA and Brazil^38,39^, a study in Saudi-Arabia revealed urban adolescents were more physically active than their rural counterparts, findings that are inconsistent with the present study^33^. Previous studies had revealed that physical activity levels of adolescents are considerably under the influence of urbanization and technological changes, including video streaming apps, and video games, particularly in urban areas and regions with higher socioeconomic status in Iran and other countries^14,29,40,41^. In addition, urbanization, the rural revolution and its consequences that include the reduction of agricultural occupations, growing access to multidimensional services, change in the structure of rural housing, motorized transport and increasing time spent on sedentary work are the major factors that lead to urban and rural lifestyle discrimination^14,24,29,42,43^.

In the current study, the prevalence of low and moderate physical activity was higher in girls than in boys (Table 2); these findings are consistent with a previous survey conducted on Iranian adolescents^44^ as well as similar surveys in America^20^, Europe^21^, China^22^, Bangladesh^45^ and Saudi-Arabia^2^. According to the 2010 WHO report, 78% of boys and 84% of girls did not meet the recommended levels of physical activity, strongly suggesting that adolescent girls are less active than boys worldwide^23^. This gender difference could possibly be attributable to the traditional structure framework of Iranian families and socio-cultural factors at the individual, school and environmental levels, which encourage boys to be more active than girls^46^. A rising body of evidence from various cultures, including Australia, Bosnia Herzegovina, Southwestern Saudi Arabia, and Oman have concurred with our results revealing that girls are more interested to spend time on sedentary activities^32,47–49^.

The current results show no statistically significant association between physical activity and body weight status in Iranian adolescents (Table 2). Controversial reports have been documented regarding the effects of physical activity on childhood obesity^50,51^. Although a number of studies have shown an inverse association between physical activity and weight status in adolescents^52–54^, further evidence does not support this association, which may be due to the multifactorial etiology and other behaviors such as daily diet^55–58^. A systematic review conducted on 44 completed RCTs and 50 ongoing studies on this topic revealed the effect of a combination of diet, physical activity and behavioral components on BMI and obesity status in a few studies^37^. Moreover, another meta-analysis on prospective observational studies assessing the association between total daily physical activity and changes in adiposity revealed no association between excessive weight gain and daily physical activity in children. They suggested a combination of a healthy diet and regular physical activity to prevent obesity^59^. Similarly, in Iran, a study conducted on school-aged children indicated physical activity was not associated with BMI^19^; which attributed to the simultaneous effects of energy intake and expenditure as components of energy balance^51,60^. Although weight status was independent of physical activity levels in the mentioned reports, exercise and physical activity had a positive impact on obesity-related consequences, including blood pressure (BP), lipids, insulin sensitivity and surrogate markers of cardiovascular health^36^.

This study is the first national report on the physical inactivity trend among Iranian adolescents residing in urban and rural areas in the Middle East, a major advantage and step in understanding and addressing this phenomenon. Nevertheless, the current study has its limitations, including gaps between 2010 and 2011 due to a lack of data collection for 2010. Also, the lack of data on the possible barriers present for physical exercise in urban and rural areas limited a more detailed interpretation of the current results. Furthermore, data collection at a national level by several interviewers and assessment of physical activity was based on participants' self-reports, which may affect its internal validity. Due to the different patterns of marginalized lifestyle and its cultural context, assessment of physical activity in sub-urban areas was not possible and that is recommended for research in future national surveys.

## Conclusion

Results of the current national report emphasize the high prevalence of physical inactivity as a nationwide problem among Iranian adolescents in both genders as well as both urban and rural residential areas. Development of effective strategies and policies to improve the status of this critical component of lifestyle among Iranian adolescents and the prevention of related disorders, particularly in girls residing in rural area, is vital and highly recommended.

## Supplementary information

Supplementary Information.

## Data Availability

The datasets used and/or analyzed during the current study are available from the corresponding authors on reasonable request.

## References

[1] Hallal PC (2012). Global physical activity levels: surveillance progress, pitfalls, and prospects. The Lancet.

[2] Al-Hazzaa HM (2011). Physical activity, sedentary behaviors and dietary habits among Saudi adolescents relative to age, gender and region. Int. J. Behav. Nutr. Phys. Act..

[3] Pearson N (2014). Associations between sedentary behaviour and physical activity in children and adolescents: a meta-analysis. Obes. Rev..

[4] (WHO)., W.H.O. Global Recommendations on Physical Activity for Health. World Health Organization. [cited 2020 25 Feb]; Available from: https://www.who.int/dietphysicalactivity/factsheet_recommendations/en/. 26180873

[5] Organization, W.H., Global Health Observatory (GHO) data. Prevalence of Insufficient Physical Activity (2016).

[6] Subhi LKA, Bose S, Ani MFA (2015). Prevalence of physically active and sedentary adolescents in 10 Eastern Mediterranean countries and its relation with age, sex, and body mass index. J. Phys. Act. Health.

[7] Fakhrzadeh H (2016). Prevalence of physical inactivity in Iran: a systematic review. J. Cardiovasc. Thorac. Res..

[8] Hovsepian S (2016). Level of physical activity and screen time among Iranian children and adolescents at the national and provincial level: The CASPIAN-IV study. Med. J. Islamic Repub. Iran.

[9] WHO., Physical activity: WHO; . 2018.

[10] Laurson KR, Lee JA, Eisenmann JC (2015). The cumulative impact of physical activity, sleep duration, and television time on adolescent obesity: 2011 youth risk behavior survey. J. Phys. Act. Health.

[11] Matin N (2017). Joint association of screen time and physical activity on self-rated health and life satisfaction in children and adolescents: the CASPIAN-IV study. Int. Health.

[12] Kelishadi R (2017). Physical inactivity and associated factors in Iranian children and adolescents: the weight disorders survey of the CASPIAN-IV study. J. Cardiovasc. Thorac. Res..

[13] Jacob C (2015). The Importance of a Life Course Approach to Health: Chronic Disease Risk from Preconception Through Adolescence and Adulthood.

[14] Janssen I (2004). Overweight and obesity in Canadian adolescents and their associations with dietary habits and physical activity patterns. J. Adolesc. Health.

[15] Strong WB (2005). Evidence based physical activity for school-age youth. J. Pediatr..

[16] Levin S (2003). Physical activity and body mass index among US adolescents: youth risk behavior survey, 1999. Arch. Pediatr. Adolesc. Med..

[17] Elinder LS, Sundblom E, Rosendahl KI (2011). Low physical activity is a predictor of thinness and low self-rated health: gender differences in a Swedish cohort. J. Adolesc. Health.

[18] Monyeki M (2015). The challenges of underweight and overweight in South African children: are we winning or losing the battle? A systematic review. Int. J. Environ. Res. Public Health.

[19] Jalali-Farahani S, Amiri P, Chin YS (2016). Are physical activity, sedentary behaviors and sleep duration associated with body mass index-for-age and health-related quality of life among high school boys and girls?. Health Qual Life Outcomes.

[20] Dumith SC (2011). Physical activity change during adolescence: a systematic review and a pooled analysis. Int. J. Epidemiol..

[21] Konstabel K (2014). Objectively measured physical activity in European children: the IDEFICS study. Int. J. Obes..

[22] Fan X, Cao Z-B (2017). Physical activity among Chinese school-aged children: national prevalence estimates from the 2016 physical activity and fitness in China—the youth study. J. Sport Health Sci..

[23] WHO, Prevalence of insufficient physical activity. WHO.

[24] Donatiello E (2013). Physical activity, adiposity and urbanization level in children: results for the Italian cohort of the IDEFICS study. Public Health.

[25] Ojiambo RM (2012). Effect of urbanization on objectively measured physical activity levels, sedentary time, and indices of adiposity in Kenyan adolescents. J. Phys. Act. Health.

[26] Pratt M (2012). The implications of megatrends in information and communication technology and transportation for changes in global physical activity. The Lancet.

[27] Patil RR (2014). Urbanization as a determinant of health: a socioepidemiological perspective. Soc. Work Public Health.

[28] Bhurosy T, Jeewon R (2014). Overweight and obesity epidemic in developing countries: a problem with diet, physical activity, or socioeconomic status?. Sci. World J..

[29] Muthuri SK (2014). Temporal trends and correlates of physical activity, sedentary behaviour, and physical fitness among school-aged children in Sub-Saharan Africa: a systematic review. Int. J. Environ. Res. Public Health.

[30] Kelishadi R (2013). Methodology and early findings of the fourth survey of childhood and adolescence surveillance and prevention of adult non-communicable disease in Iran: the CASPIAN-IV study. Int. J. Prev. Med..

[31] Esteghamati A (2016). Awareness, treatment and control of pre-hypertension, and hypertension among adults in Iran. Arch. Iran. Med..

[32] Esteghamati A (2011). Physical activity in Iran: results of the third national surveillance of risk factors of non-communicable diseases (SuRFNCD-2007). J. Phys. Act. Health.

[33] Khosla T, Lowe C (1967). Indices of obesity derived from body weight and height. Br. J. Prev. Soc. Med..

[34] StataCorp. 2015. Stata Statistical Software: Release 14. College Station, T.S.L.

[35] Angoorani P (2018). The association of parental obesity with physical activity and sedentary behaviors of their children: the CASPIAN-V study. J. Pediatr..

[36] Pälve KS (2014). Association of physical activity in childhood and early adulthood with carotid artery elasticity 21 years later: the cardiovascular risk in Young Finns Study. J. Am. Heart Assoc..

[37] Al‐Khudairy, L. *et al.* Diet, physical activity and behavioural interventions for the treatment of overweight or obese adolescents aged 12 to 17 years. Cochrane Database Syst. Rev. **6** (2017) 10.1002/14651858.CD012691 PMC6481371 28639320

[38] Johnson Iii JA, Johnson AM (2015). Urban-rural differences in childhood and adolescent obesity in the United States: a systematic review and meta-analysis. Child. Obes.

[39] Regis MF (2016). Urban versus rural lifestyle in adolescents: associations between environment, physical activity levels and sedentary behavior. Einstein (São Paulo).

[40] Kelishadi R (2016). Socioeconomic disparities in dietary and physical activity habits of Iranian children and adolescents: the CASPIAN-IV study. Arch. Iran. Med..

[41] Singh AP, Misra G (2012). Adolescent lifestyle in India: Prevalence of risk and promotive factors of health. Psychol. Dev. Soc..

[42] Bassett DR (2015). Trends in physical activity and sedentary behaviors of United States youth. J. Phys. Act. Health.

[43] Soltani Moghadas R (2013). Transmission of physical texture of nearby metropolitan villages (case study: rural houses of Torghabeh Dehestan). J. Phys. Dev. Plan..

[44] Heshmat R (2016). Joint association of screen time and physical activity with cardiometabolic risk factors in a national sample of Iranian adolescents: the CASPIANIII study. PLoS ONE.

[45] Khan A, Burton N, Trost S (2017). Patterns and correlates of physical activity in adolescents in Dhaka city, Bangladesh. Public health.

[46] Shokrvash B (2013). Correlates of physical activity in adolescence: a study from a developing country. Global Health Action.

[47] Jandrić S (2010). Differences between boys and girls in terms of physical activity. Facta Univ. Ser. Phys. Educ. Sport.

[48] Telford RM (2016). Why are girls less physically active than boys? Findings from the LOOK longitudinal study. PLoS ONE.

[49] Esteghamati A (2009). Association between physical activity and insulin resistance in Iranian adults: national surveillance of risk factors of non-communicable diseases (SuRFNCD-2007). Prev. Med..

[50] McMurray R (1995). Childhood obesity elevates blood pressure and total cholesterol independent of physical activity. Int. J. Obes..

[51] Luke A, Cooper RS (2013). Physical activity does not influence obesity risk: time to clarify the public health message. Int. J. Epidemiol..

[52] Belcher BR (2010). Physical activity in US youth: Impact of race/ethnicity, age, gender, & weight status. Med. Sci. Sports Exerc..

[53] Riddoch CJ (2009). Prospective associations between objective measures of physical activity and fat mass in 12–14 year old children: the Avon longitudinal study of parents and children (ALSPAC). BMJ.

[54] Ness AR (2007). Objectively measured physical activity and fat mass in a large cohort of children. PLoS Med..

[55] Stevens J (2007). Objectively assessed associations between physical activity and body composition in middle-school girls: the trial of activity for adolescent girls. Am. J. Epidemiol..

[56] van Sluijs, E. *et al*. The Association between Change in Physical Activity and Weight during Adolescence (2019).

[57] McMurray RG (2008). Influence of physical activity on change in weight status as children become adolescents. Int. J. Pediatric Obes..

[58] Naseri P (2020). Longitudinal association between body mass index and physical activity among adolescents with different parental risk: a parallel latent growth curve modeling approach. Int. J. Behav. Nutr. Phys. Act..

[59] Wilks DC (2011). Objectively measured physical activity and fat mass in children: a bias-adjusted meta-analysis of prospective studies. PLoS ONE.

[60] Hill JO, Wyatt HR, Peters JC (2012). Energy balance and obesity. Circulation.

