# Supplementary material for: Trends of low physical activity among Iranian adolescents across urban and rural areas during 2006–2011
Source: Sci Rep. 2020 Dec 7;10:21318. doi: 10.1038/s41598-020-78048-0 (PMC7721745; doi:10.1038/s41598-020-78048-0)
Supplement: Supplementary file 1 — Supplementary Information. [file 41598_2020_78048_MOESM1_ESM.docx]

**Title page:**

**Trends of low physical activity among Iranian adolescents across urban and rural areas during 2006-2011**

**Authors' names:**

Parisa Amiri^1*^, Parisa Naseri^1,2*^, Golnaz Vahedi-Notash^1^, Sara Jalali-Farahani^1^, Yadollah Mehrabi^3^, Najmeh Hamzavi-Zarghani^1^, Fereidoun Azizi^4^, Farzad Hadaegh^5^, Davood Khalili^5^

**Affiliations:**

1. Research Center for Social Determinants of Health, Research Institute for Endocrine Sciences, Shahid Beheshti University of Medical Sciences, Tehran, Iran
2. Department of Biostatistics, Faculty of Paramedical Sciences, Shahid Beheshti University of Medical Sciences, Tehran, Iran
3. Department of Epidemiology, School of Public Health and Safety, Shahid Beheshti University of Medical Sciences, Tehran, Iran
4. Endocrine Research Center, Research Institute for Endocrine Sciences, Shahid Beheshti University of Medical Sciences, Tehran, Iran
5. Prevention of Metabolic Disorders Research Center, Research Institute for Endocrine Sciences, Shahid Beheshti University of Medical Sciences, Tehran, Iran

***The authors had an equal contribution.**

**Running title:** Trends of low physical activity among Iranian adolescents

**Word count:** 3457

**Corresponding author:**

**Davood Khalili, MD, PhD**

[Prevention of Metabolic Disorders Research Center](https://www.researchgate.net/institution/Shahid_Beheshti_University_of_Medical_Sciences),

Research Institute for Endocrine Sciences,

Shahid Beheshti University of Medical Sciences,

P.O.Box: 19395-4763, Tehran, I. R. Iran,

Tel: +98 21 2409309, Fax: +98 21 2402463,

Email: [dkhalili@endocrine.ac.ir](mailto:dkhalili@endocrine.ac.ir)

**Table 1-Appendix**. Sex-specific characteristics of adolescents aged 15-19 years: SuRFNCD2006- 2011.

|  | **Girls** | | | | | **Boys** | | | | |
| --- | --- | --- | --- | --- | --- | --- | --- | --- | --- | --- |
|  | **2006**  **(n=1188)** | **2007**  **(n=1285)** | **2008**  **(n=1316)** | **2009**  **(n=1309)** | **2011**  **(n=490)** | **2006**  **(n=1407)** | **2007**  **(n=1612)** | **2008**  **(n=1634)** | **2009**  **(n=1518)** | **2011**  **(n=419)** |
| **Age (years)** | 17.79±0.03 | 17.67±0.03 | 17.68±0.03 | 17.56±0.04 | 17.69±0.06 | 17.77±0.03 | 17.50±0.03 | 17.46±0.03 | 17.61±0.03 | 17.70±0.07 |
| **Area** n(%) |  | | | | | | | | | |
| Urban | 693(58.3) | 738(57.4) | 800(60.8) | 709(54.2) | 327(66.7) | 887(63) | 950(58.9) | 997(61) | 814 (53.6) | 274(65.4) |
| Rural | 495(41.7) | 547(42.6) | 516(39.2) | 600 (45.8) | 163(33.3) | 520(37) | 662(41.1) | 637(39) | 704(46.4) | 145(34.6) |
| **Physical activity** (%±SE**)** |  | | | | | | | | | |
| High | 26.84±1.32 | 22.90±1.23 | 24.02±1.24 | 21.80±1.23 | 21.58±2.12 | 70.43±1.21 | 67.31±1.21 | 64.14±1.20 | 61.87±1.31 | 59.86±2.52 |
| Moderate | 42.50±1.52 | 40.76±1.50 | 34.64±1.43 | 37.07±1.50 | 32.23±2.36 | 20.03±1.06 | 21.63±1.07 | 21.69±1.05 | 23.89±1.17 | 23.41±2.20 |
| Low | 30.64±1.41 | 36.32±1.45 | 41.33±1.47 | 41.11±1.51 | 46.18±2.49 | 9.53±0.79 | 11.05±0.81 | 14.16±0.89 | 14.23±0.97 | 16.72±1.87 |
| **General obesity (%**±SE**)** |  | | | | | | | | | |
| Obese | 20.82±1.18 | 20.65±1.15 | 20.10±1.12 | 23.33±1.21 | 23.68±1.90 | 17.22±1.00 | 18.29±0.97 | 18.58±0.97 | 18.95±1.05 | 17.14±1.83 |
| Non-obese | 79.17±1.18 | 79.34±1.15 | 79.89±1.12 | 76.66±1.21 | 76.31±1.90 | 82.77±1.00 | 81.70±0.97 | 81.41±0.97 | 81.04±1.05 | 82.85±1.83 |

Age, Physical activity and weight status values are Mean± SEM. Variables (except residential area) are standardized according to residential area.

Non-obese: underweight/normal

Obese: overweight/obese

**Table 2- Appendix.** Unadjusted prevalence % (95%CI) and trend of low physical activity in Iranian adolescents, aged 15-19 based on sex and residential area: 2006-2011.

|  | **2006** | **2007** | **2008** | | **2009** | **2011** | **P for trend** |
| --- | --- | --- | --- | --- | --- | --- | --- |
| **Girls** | | | | | | | |
| **Urban** | | | | | | | |
| low | 30.37(25.55-35.66) | 38.75(33.61-44.14) | 43.64(38.52-48.91) | | 44.02(38.52-49.68) | 46.34(38.17-54.71) | 0.05 |
| **Rural** | | | | | | | |
| low | 31.20(25.62-37.39) | 31.25(25.81-37.25) | 36.34(30.28-42.86) | | 35.09(29.63-40.98) | 45.83(33-885.58.30) | 0.02 |
| **Boys** | | | | | | | |
| **Urban** | | | | | | | |
| low | 10.24(7.71-13.48) | 11.71(9.05-15.03) | 16.84(13.73-20.49) | | 17.06(13.60-21.18) | 17.57(12.00-24.99) | 0.05 |
| **Rural** | | | | | | | |
| low | 8.07(5.31-12.07) | 9.71(6.92-13.46) | | 8.78(6.08-12.51) | 8.63(6.06-12.14) | 15.03(8.21-25.91) | 0.09 |
